# The prognostic impact of severe grade immune checkpoint inhibitor related pneumonitis in non-small cell lung cancer patients

**DOI:** 10.3389/fonc.2024.1372532

**Published:** 2024-06-25

**Authors:** Ni Sun, Ru Li, Haiyi Deng, Qingyang Li, Jiaxi Deng, Yue Zhu, Wenwei Mo, Wenhui Guan, Minjuan Hu, Ming Liu, Xiaohong Xie, Xinqing Lin, Chengzhi Zhou

**Affiliations:** ^1^ Guangzhou Medical University, Guangzhou, Guangdong, China; ^2^ State Key Laboratory of Respiratory Disease, National Clinical Research Center for Respiratory Disease, National Center for Respiratory Medicine, Department of Pulmonary and Critical Care Medicine-Section 5, Guangzhou Institute of Respiratory Health, the First Affiliated Hospital of Guangzhou Medical University, Guangzhou, Guangdong, China; ^3^ Henan University, Kaifeng, Henan, China

**Keywords:** immune checkpoint inhibitor, pneumonitis, non-small cell lung cancer, prognosis, cause of death

## Abstract

**Objective:**

To compare the prognostic differences between non-small cell lung cancer (NSCLC) patients with mild and severe checkpoint inhibitor-associated pneumonitis (CIP), and explore the causes of death and prognostic risk factors in NSCLC patients with severe CIP.

**Methods:**

A retrospective study of a cohort of 116 patients with unresectable stage III or IV NSCLC with any grade CIP from April 2016 to August 2022 were conducted. To analyze the clinical characteristics of patients with different CIP grades, patients were divided into mild CIP group (grade 1-2, n=49) and severe CIP group (grade 3-5, n=67) according to the grade of CIP. To explore the OS-related risk factors in the severe CIP group, the patients were divided into a good prognosis (GP) group (≥ median OS, n=30) and a poor prognosis (PP) group (< median OS, n=37) based on whether their overall survival (OS) were greater than median OS. Baseline clinical and laboratory data were collected for analysis.

**Results:**

The median OS of all NSCLC patients combined with CIP was 11.4 months (95%CI, 8.070–16.100), The median OS for mild CIP and severe CIP was 22.1 months and 4.4 months respectively (HR=3.076, 95%CI, 1.904-4.970, P<0.0001). The results showed that the most common cause of death among severe CIP patients in the PP group was CIP and the most common cause in the GP group was tumor. The univariate regression analysis showed that suspension of antitumor therapy was a risk factor for poor prognosis (OR=3.598, 95%CI, 1.307-9.905, *p*=0.013). The multivariate logistic regression analysis showed that suspension of anti-tumor therapy (OR=4.24, 95%CI, 1.067-16.915, *p*=0.040) and elevated KL-6 (OR=1.002, 95%CI, 1.001-1.002, *p*<0.001) were independent risk factors for poor prognosis.

**Conclusion:**

In conclusion, patients with severe CIP had a poor prognosis, especially those with elevated KL-6, and the main cause of death is immune checkpoint inhibitor-associated pneumonitis complicated with infection. In addition, anti-tumor therapy for severe CIP patients should be resumed in time and should not be delayed for too long.

## Introduction

Over the past decade, China has experienced the highest incidence and mortality rates of lung cancer globally, with non-small cell lung cancer (NSCLC) comprising approximately 80% of all diagnosed cases (1, 2). With the advancement of medical research, in addition to conventional chemotherapy, progress in cancer genomics has provided novel avenues for targeted therapy in lung cancer patients harboring driver gene mutations (3–7). However, for patients lacking driver gene mutations and those resistant to chemotherapy and targeted therapies, cancer immunology and immunotherapy offer a fresh perspective in cancer treatment. Use of immune checkpoint inhibitors (ICIs), including antibodies against programmed cell death protein 1 (PD-1) or its ligand PD-L1, has significantly improved overall survival (OS) in NSCLC (8). ICIs mainly mediates the destruction of cancer cells by activating the antitumor function of T cells; however, the deinhibition of T cell function by ICIs can lead to a series of organ-specific inflammatory side effects known as immune-related adverse events (irAEs). These irAEs result from unintended effects of the ICIs-mediated activation of the immune system and can occur in any organ system. Checkpoint inhibitor-associated pneumonitis (CIP) induced by ICIs is considered one of the more serious irAEs (9). Early data from clinical trials and other studies reported CIP in only 3% to 7% of patients, but more recently this phenomenon was reported to occur in nearly 20% of patients with NSCLC who received one or more of these agents outside of clinical trials (10). Therefore, clinicians need to pay more attention to this serious irAE. CIP has a more frequent occurrence and a faster rate of onset in NSCLC compared to other types of cancer (10). Therefore, clinicians need to pay more attention to this serious irAE.

The development of irAEs, including CIP, was considered a good predictive factor for the efficacy of ICIs treatment. Besides, patients who experienced an irAE had significantly longer progression free survival (PFS) and OS compared with those without irAEs (2, 11). However, some other research found that CIP was a serious complication with a poor prognosis in patients with NSCLC undergoing ICIs therapy, and the efficacy of ICIs was significantly worst in patients with severe CIP than in those without severe CIP (12). However, few articles have focused on the prognosis, cause of death, and survival risk factors of severe CIP. Understanding the prognosis of CIP can help us to better manage it.

Herein, the objective of this study is to compare the differences in prognosis between NSCLC patients with mild and severe CIP and to identify the related causes of death and influencing factors of poor prognosis in patients with severe CIP.

## Materials and methods

### Subjects

A retrospective study of a cohort of 116 patients with unresectable stage III or IV NSCLC with any grade CIP were conducted. All subjects were enrolled from the First Affiliated Hospital of Guangzhou Medical University for analysis from April 2016 to August 2022. To analyze the clinical characteristics of patients with different CIP grades, patients were divided into mild CIP group (grade 1-2, n=49) and severe CIP group (grade 3-5, n=67) according to the grade of CIP. To explore the OS-related risk factors in the severe CIP group, the patients were divided into a good prognosis (GP) group (≥ median OS, n=30) and a poor prognosis (PP) group (< median OS, n=37) based on whether their OS were greater than median OS. This study protocol was formulated in accordance with the requirements of the Declaration of Helsinki of the World Medical Association. This study was approved by the local Ethics Committee of the First Affiliated Hospital of Guangzhou Medical University (2005L01528).

### Inclusion and exclusion criteria

Inclusion criteria: 1. Patients over 18 years old; 2. Patients with pathologically confirmed advanced (inoperable stage IIIB-IV) primary lung carcinoma with at least one measurable lesion that meets RECIST v1.1 criteria; 3. ICIs treatment was carried out in the clinical practice of the patient. 4. The diagnosis of CIP was established by a panel consisting of two seasoned pulmonologists and a chest radiologist, adhering to the standards outlined by the National Comprehensive Cancer Network, the American Society for Clinical Oncology, and the European Society for Medical Oncology (11–13).

Exclusion criteria: 1. Patients who have experienced tuberculosis, bacterial or fungal infection before ICI treatment (To exclude influence of these diseases on cytokines (14)); 2. Patients with incomplete clinical data. 3. Patients with clinical symptoms or diseases of the heart that are not well controlled, such as: a. NYHA grade 2 or higher heart failure; b. Unstable angina pectoris; c. Myocardial infarction within 1 year; d. Clinically significant supraventricular or ventricular arrhythmias requiring treatment or intervention.

### ICIs treatment protocol

All ICIs therapeutic measurements shall apply the standard measurements specified in the National Comprehensive Cancer Network (NCCN) guidelines (15). Patients were given Nivolumab 3 mg/kg, Pembrolizumab 200 mg, Atezolizumab 1200 mg, or other programmed cell death protein-1 (PD-1)/PD-L1 inhibitors according to the requirements of clinical trials. Every 2 or 3 weeks until disease progression or unacceptable ICIs related toxicity was confirmed. All patients were treated with single ICIs.

### Data collection

The medical records were reviewed, and the following basic characteristics of patients were collected: age, gender, body mass index (BMI), smoking history, Eastern Cooperative Oncology Group performance status (ECOG PS), treatment line, TNM stage, histologic classification, coexisting conditions, etc. The histologic classification of NSCLC was based on the World Health Organization criteria (2015 version) (16). The baseline time point was the time point at which CIP was diagnosed. The coexisting cardiovascular disease included specific diseases of hypertension, coronary artery disease and arrhythmia. The history of prior lung disease encompasses emphysema chronic obstructive pulmonary disease (COPD), obstructive pneumonitis and Interstitial lung disease.

We defined suspension of antitumor therapy as discontinuation of antitumor therapy after CIP occurred for more than 5 treatment cycles.

The laboratory data on the patient were also collected at the time of diagnosis of CIP, including interleukin-2 (IL-2), interleukin-4 (IL-4), interleukin-6 (IL-6), interleukin-10 (IL-10), tumor necrosis factor (TNF-α), interferon-gamma receptor (IFN -γ), high sensitivity C reactive protein (hsCRP), lactate dehydrogenase (LDH), albumin (ALB), neutrophil (NEUT), absolute lymphocyte count (ALC), platelet (PLT), neutrophil-to-lymphocyte (NLR), platelet lymphocyte ratio (PLR), D-Dimer and human sialylated carbohydrate antigen 6 (KL-6).

In addition, the specific treatment protocols and follow-up results of patients were also collected. The OS was determined from the date of confirmed CIP to death or last follow-up evaluation.

### Evaluation and treatment of CIP

CIP was defined as the emergence of new infiltrates on thoracic imaging, accompanied by or without clinical manifestations such as cough, shortness of breath, or wheezing, all of which were deemed likely to be induced by ICIs. Other potential etiologies were thoroughly excluded from the diagnostic considerations. At the same time, a number of other causes, including lung infection or tumor progression, need to be ruled out by bronchoalveolar perfusion culture, sputum culture, echocardiography, and laboratory tests (routine blood tests, procalcitonin, tumor markers, arterial gas analysis, serous D-dimer, and brain natriuretic peptide, etc.) before diagnosis was made.

### Causes of death

The causes of death of patients in this study were divided into the following three categories: 1. Death directly caused by CIP; 2. Death caused by tumors, such as tumor progression, tumor emergencies, or complications during tumor treatment; 3. Other reasons, such as other irAEs or other underlying diseases. The telephone follow-up was performed at bi-monthly intervals from the onset of CIP until loss to follow-up or death through August 2022.

### Statistical analysis

The measurement data were expressed by medians and ranges. Associations between continuous variables were by the Wilcoxon-Mann-Whitney test or the Kruskal-Wallis test, when appropriate. Categorical variables will be compared by chi-square test (χ2) or Fisher’s exact test in terms of frequency, and described by frequency and percentage (%). Kaplan–Meier estimates and the log-rank test were used to evaluate the indicator of OS. In addition, the logistic regression models were performed to examine which risk factors were independently associated with OS. For each test, two-sided P values of <0.05 were considered statistically significant. All analyses were conducted using R Studio (4.2.2) and Jamovi (2.3.13). The statistical significance is set to 2 sides with *p*=0.05.

## Results

### Baseline clinical characteristics and comparison of OS between mild CIP group and severe CIP group

**Figure 1:**
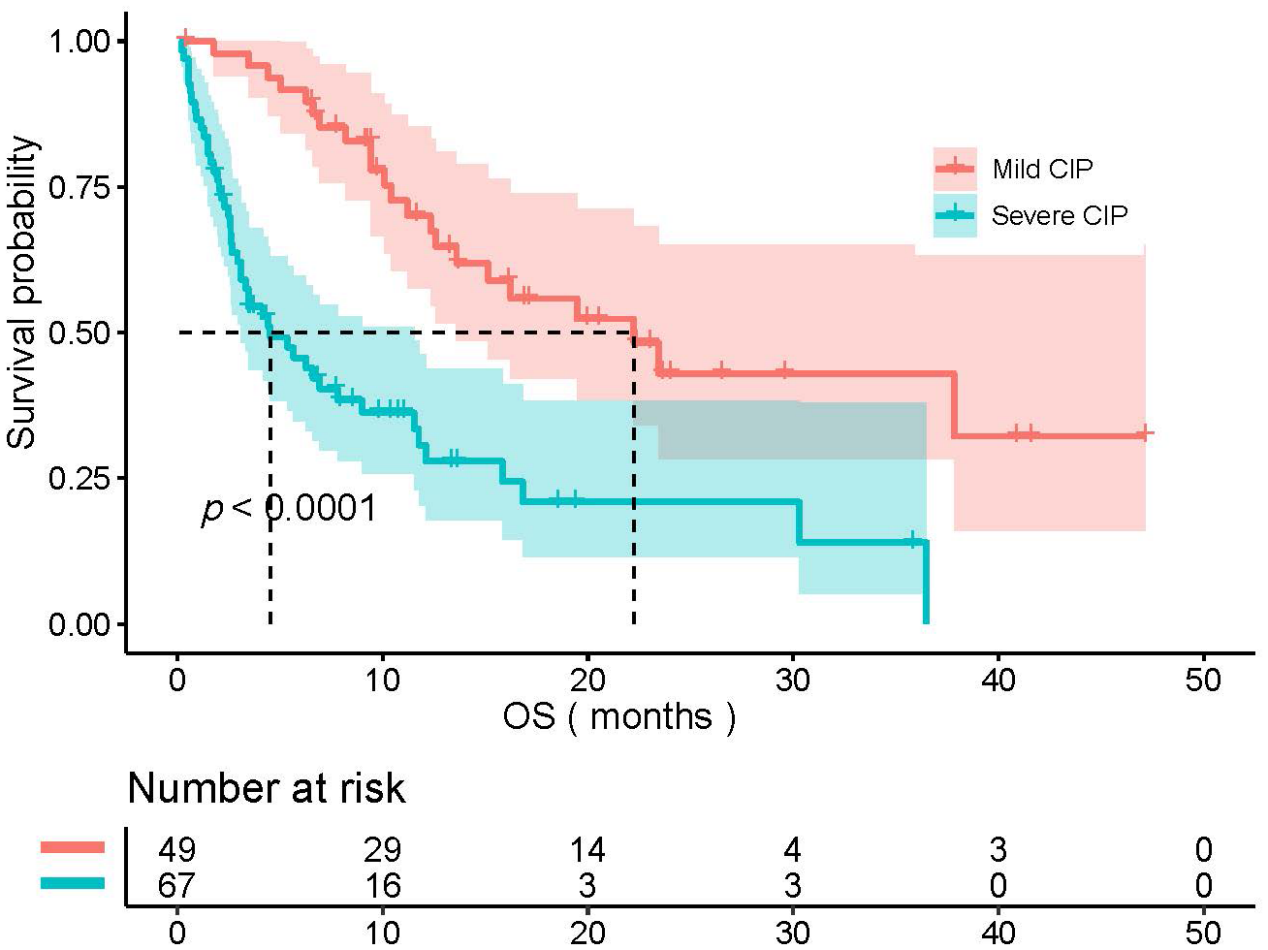
The comparison of OS between mild CIP group and severe CIP group. OS, overall survival; CIP, checkpoint inhibitor pneumonitis.

A total of 116 patients were included. The mild CIP group consisted of 49 patients with 44 males and 5 females. The severe CIP group consisted of 67 patients with 59 males and 8 females. In the severe CIP group, the proportion of ECOG score≥2 (*p*<0.001) and the proportion of NON-SCC (*p*=0.009) were significantly higher than those in the mild CIP group. Other baseline data were not significantly different between the two groups and were presented in **Table 1**. The median OS (mOS) of all NSCLC patients combined with CIP was 11.4 months (95%CI,8.070–16.100). The median OS for mild CIP and severe CIP was 22.1 months and 4.4 months respectively (HR=3.076, 95%CI, 1.904-4.970, *p*<0.0001). The data of OS was shown in **Figure 1**. Patients in mild CIP group had significantly longer OS than those in severe CIP group (*p*<0.0001).

**Table 1:** Baseline characteristics of patients.

Variables	mild CIP group (n=49)	severe CIP group (n=67)	Overall (n=116)	*p*-value
**Gender, n (%)**				0.770
Male	44 (89.8%)	59 (88.1%)	103 (88.8%)	
Female	5 (10.2%)	8 (11.9%)	13 (11.2%)	
**Age (years)**				0.272
≥65	27 (55.1%)	30 (44.8%)	57 (49.1%)	
<65	22 (44.9%)	37 (55.2%)	59 (50.9%)	
**Smoking history**				0.626
Ever smoking	30 (61.2%)	38 (56.7%)	68 (58.6%)	
Never smoking	19 (38.8%)	29 (43.3%)	48 (41.4%)	
**ECOG PS**				< 0.001
0-1	45 (91.8%)	44 (65.7%)	89 (76.7%)	
≥2	4 (8.2%)	23 (34.3%)	27 (23.3%)	
**Histological**				0.009
Squamous	31 (63.3%)	26 (38.8%)	57 (49.1%)	
Adenocarcinoma	18 (36.7%)	41 (61.2%)	59 (50.9%)	
**Stage**				0.663
3	18 (36.7%)	22 (32.8%)	40 (34.5%)	
4	31 (63.3%)	45 (67.2%)	76 (65.5%)	
**Lines of ICIs**				0.735
First line	39 (79.6%)	55 (82.1%)	94 (81.0%)	
≥Second line	10 (20.4%)	12 (17.9%)	22 (19.0%)	
**PD-L1 expression**				0.389
Undetected	22 (44.9%)	38 (56.7%)	38 (56.7%)	
Positive	12 (24.5%)	15 (22.4%)	27 (23.3%)	
Negative	15 (30.6%)	14 (20.9%)	29 (25.0%)	
**Distant metastasis**	25 (51.0%)	37 (55.2%)	62 (53.4%)	0.654
Coexisting conditions				
Combine other irAEs	8 (16.3%)	9 (13.4%)	17 (14.7%)	0.841
Cardiovascular disease	11 (22.4%)	14 (20.9%)	25 (21.6%)	0.841
History of prior lung disease	33 (67.3%)	38 (56.7%)	71 (61.2%)	0.246

CIP, checkpoint inhibitor related pneumonitis; ECOG, Eastern Cooperative Oncology Group performance status; irAEs, Immune-related adverse events; SCC, Squamous cell carcinoma; COPD, Chronic Obstructive Pulmonary Disease. The history of prior lung disease encompasses emphysema, COPD, obstructive pneumonitis, and Interstitial lung disease.

### Clinical characteristics of patients in severe CIP group

A subgroup analysis of the clinical characteristics of 67 patients in the severe CIP group in this section was further conducted. Based on whether the OS was greater than the median OS, patients in the severe CIP group were divided into the GP group (greater than the median OS) and the PP group (less than the median OS). The baseline clinical characteristics of the severe CIP group were presented in **Table 2**. In the severe CIP group, 59 (88.1%) patients were male, 38 (56.7%) had a history of smoking, 64 (95.5%) had an ECOG ≤ 2, and 41 (61.2%) had a histological type of lung adenocarcinoma. 55 (82.1%) patients received ICIs treatment at the time of first-line treatment, and 15 (22.4%) patients had positive PD-L1 expression. There were no significant differences in baseline clinical characteristics between the two groups except that the KL-6 levels exhibited a significant difference between the GP group and the PP group, with a statistical significance of P < 0.001. The laboratory results of the severe CIP group were presented in **Table 3**. There were no significant differences in laboratory data between the two groups (all *p*>0.05).

**Table 2:** Baseline characteristics of patients in severe CIP group.

Variables	PP group (n=37)	GP group (n=30)	Overall (n=67)	*p*-value
**Gender, n (%)**				0.283
Male	34 (91.9%)	25 (83.3%)	59 (88.1%)	
Female	3 (8.1%)	5 (16.7%)	8 (11.9%)	
**Age (years)**				0.205
≥65	14 (37.8%)	16 (53.3%)	30 (44.8%)	
<65	23 (62.2%)	14 (46.7%)	37 (55.2%)	
**BMI (kg/m^2^)**	19.8 (15.4, 26.7)	20.8 (16.0, 27.6)	20.4 (15.4, 27.6)	0.070
**Smoking history**				0.994
Ever smoking	21 (56.8%)	17 (56.7%)	38 (56.7%)	
Never smoking	16 (43.2%)	13 (43.3%)	29 (43.3%)	
**ECOG PS**				0.683
≤2	35 (94.6%)	29 (96.7%)	64 (95.5%)	
3	2 (5.4%)	1 (3.3%)	3 (4.5%)	
**Histological**				0.857
Squamous	14 (37.8%)	12 (40.0%)	26 (38.8%)	
Adenocarcinoma	23 (62.2%)	18 (60.0%)	41 (61.2%)	
**Agent**				0.829
PD-1 inhibitors	35 (94.6%)	28 (93.3%)	63 (94.0%)	
PD-L1 inhibitors	2 (5.4%)	2 (6.7%)	4 (6.0%)	
**Lines of ICIs**				0.128
First line	28 (75.7%)	27 (90.0%)	55 (82.1%)	
≥Second line	9 (24.3%)	3 (10.0%)	12 (17.9%)	
**PD-L1 expression**				0.362
Undetected	20 (54.1%)	18 (60.0%)	38 (56.7%)	
Positive	7 (18.9%)	8 (26.7%)	15 (22.4%)	
Negative	10 (27.0%)	4 (13.3%)	14 (20.9%)	
**Distant metastasis**	19 (51.4%)	18 (60.0%)	37 (55.2%)	0.480
Coexisting conditions				
**Combine other irAEs**	6 (16.2%)	3 (10.0%)	9 (13.4%)	0.458
**Cardiovascular disease**	9 (24.3%)	5 (16.7%)	14 (20.9%)	0.443
History of prior lung disease				
COPD	9 (24.3%)	4 (13.3%)	13 (19.4%)	0.258
Emphysema	12 (32.4%)	6 (20.0%)	18 (26.9%)	0.254
**History of acute radiation pneumonitis**	0 (0%)	2 (6.7%)	2 (3.0%)	0.111

Abbreviation: CIP, checkpoint inhibitor related pneumonitis; PP, poor prognosis; GP, good prognosis; BMI, Body Mass Index; ECOG PS, Eastern Cooperative Oncology Group performance status; ICI, immune checkpoint inhibitor; PD-1, programmed cell death protein-1; irAEs, Immune-related adverse events; COPD, Chronic obstructive pulmonary disease.

**Table 3:** The laboratory data of patients in severe CIP group (median (range)).

Variables	PP group (n=37)	GP group (n=30)	Overall (n=67)	*p*-value
**IL-2, pg/mL**	1.23 (0.02, 308)	1.12 (0.10, 38.3)	1.18 (0.02, 308)	0.410
**IL-4, pg/mL**	1.80 (0.03, 5.50)	1.28 (0.03, 35.6)	1.67 (0.03, 35.6)	0.188
**IL-6, pg/mL**	36.1 (1.09, 5000)	44.3 (1.91, 26900)	38.2 (1.09, 26900)	0.719
**IL-10, pg/mL**	5.12 (0.47, 73.7)	5.06 (1.35, 98.8)	5.12 (0.47, 98.8)	0.729
**TNF, pg/mL**	1.89 (0.52, 13.0)	1.50 (0.42, 134)	1.70 (0.42, 134)	0.427
**INF, pg/mL**	1.68 (0.22, 203)	1.30 (0.51, 45.5)	1.48 (0.22, 203)	0.438
**hsCRP**	71.2 (3.84, 187)	30.9 (1.98, 365)	44.9 (1.98, 365)	0.136
**LDH, U/L**	342 (0.90, 1640)	302 (168, 695)	307 (0.90, 1640)	0.457
**ALB, g/L**	31.3 (22.3, 39.3)	31.8 (23.4, 42.9)	31.4 (22.3, 42.9)	0.650
**NEUT, K/μL**	9.60 (3.00, 41.1)	8.62 (0.40, 82.5)	8.80 (0.40, 82.5)	0.772
**ALC, K/μL**	0.80 (0.20, 6.70)	0.95 (0.30, 11.0)	0.90 (0.20, 11.0)	0.277
**PLT, K/μL**	230 (27.0, 739)	269 (79.0, 640)	253 (27.0, 739)	0.449
**NLR**	12.6 (1.15, 54.5)	9.56 (0.64, 135)	11.7 (0.64, 135)	0.198
**PLR**	334 (16.3, 1043)	337 (20.6, 1053)	337 (16.3, 1053)	0.569
**D-Dimer**	2140 (586, 10000)	2310 (420, 10000)	2160 (420, 10000)	0.541
**KL-6, U/ml**	638 (111, 2172)	1670 (350, 8009)	1102 (111, 8009)	**<0.001**

CIP, checkpoint inhibitor related pneumonitis; PP, poor prognosis; GP, good prognosis; IL-2 interleukin-2; TNF-α, tumor necrosis factor; IFN –γ, interferon-gamma receptor; hsCRP, high sensitivity C reactive protein; LDH, lactate dehydrogenase; ALB, albumin; NEUT, neutrophil; ALC, absolute lymphocyte count; PLT, platelet; NLR, neutro-phil-to-lymphocyte; PLR, platelet lymphocyte ratio; KL-6, human sialylated carbohydrate antigen 6.

### Causes of death in patients in severe CIP group

**Figure 2:**
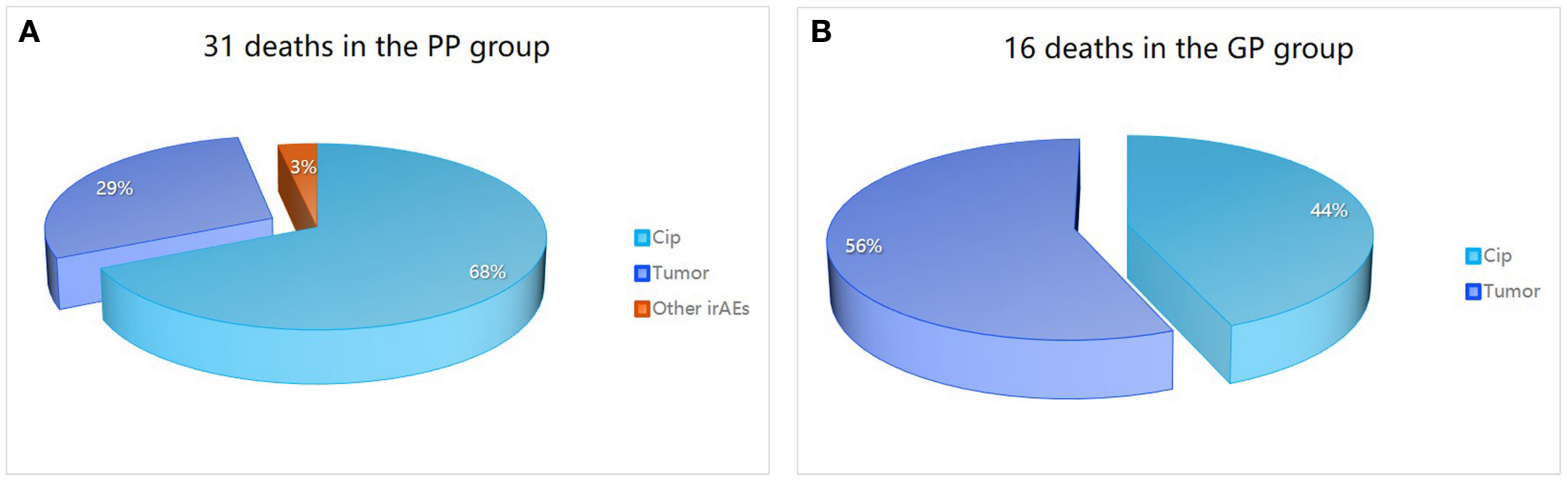
Causes of death in patients in severe CIP group. Causes of death in the PP group **(A)** is different from that in the GP group **(B)**. PP, poor prognosis; GP, good prognosis; CIP, checkpoint inhibitor pneumonitis; irAEs, immune-related adverse events.

**Figure 3:**
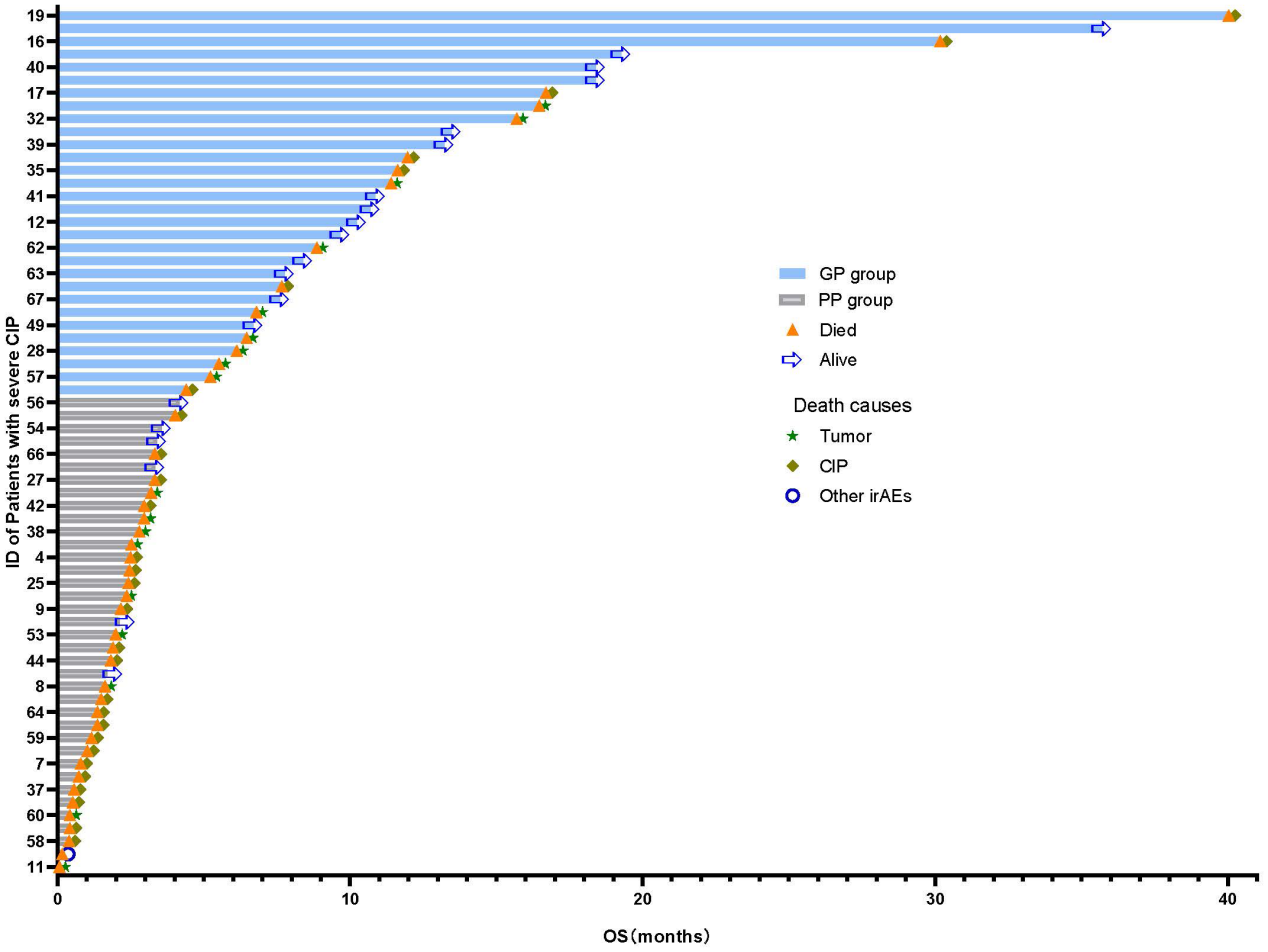
Swimmer plots of the disease course of severe immune checkpoint inhibitor related pneumonitis patients. In the GP group, the overall survival surpasses the median overall survival of 4.4 months observed among severe CIP. In the PP group, the overall survival is shorter than the median overall survival of 4.4 months observed among severe CIP. PP, poor prognosis; GP, good prognosis, CIP, checkpoint inhibitor pneumonitis.

By the end of follow-up, a total of 47 patients in the severe CIP group had died. Among severe CIP patients, 28 (59.57%) died due to CIP, with 7 (43.75%) in the GP group and 21 (67.74%) in the PP group (P=0.112). Additionally, 18 (38.30%) patients died due to tumor, with 9 (56.25%) in the GP group and 9 (29.03%) in the PP group (*p* = 0.069). One patient (3.23%) in the PP group died from immune myocarditis. The chi-square test results for the analysis of causes of death in the severe group are shown in **Table 4** and **Figure 2**. Swimmer plots shows the disease course of all severe immune checkpoint inhibitor related pneumonitis patients (**Figure 3**).

**Table 4:** Chi-square analysis of the causes of death in the severe CIP group.

Causes of death	Total (n = 47)	GP (n = 16)	PP (n = 31)	p-value
Tumor,(n%)	18 (38.30)	9 (56.25)	9 (29.03)	0.069
CIP,n(%)	28 (59.57)	7 (43.75)	21 (67.74)	0.112
Other irAEs, n(%)	1 (2.13)	0 (0.00)	1 (3.23)	1.000

GP, good prognosis; PP, poor prognosis; CIP, checkpoint inhibitor pneumonitis; irAEs, immune related adverse events.

### Risk factors for poor prognosis in patients with severe CIP

The univariate and multivariate logistic regression analysis were performed to explore risk factors for poor outcomes in patients with severe CIP. In univariate regression analysis, the dependent variable was whether the patient was in the PP group, and the independent variable was the baseline clinical characteristics and laboratory data of the patient. Univariate regression analysis showed that suspension of antitumor therapy (OR=3.598, 95%CI, 1.307-9.905, *p*=0.013) and KL-6(OR=1.001, 95%CI, 1.001-1.002, *p*<0 .001) were risk factors for poor prognosis. And no other statistical difference was found in other independent variables (**Table 5**). According to the results of the literature review, we included the previously reported independent variables of high risk, such as IL-6, IL-10, LDH, KL-6, smoking history and suspension of anti-tumor therapy into the multivariate regression analysis (15, 17–20). Multivariate Logistic regression analysis showed that suspension of anti-tumor therapy (OR=4.247, 95%CI, 1.067-16.915, *p*=0.040) and KL-6 (OR=1.002,95%CI, 1.001-1.002, *p*<0 .001) were independent risk factors for poor prognosis (**Table 6**).

**Table 5:** The univariate logistic regression analysis of risk factors for poor prognosis in patients with severe CIP.

Variables	OR (95% CI)	*p*-value
**Gender (male vs female)**	0.441 (0.096-2.020)	0.292
**Age (≥ 65 vs < 65)**	1.878 (0.706-4.991)	0.207
**BMI**	1.152(0.968-1.371)	0.110
**Smoking history (Yes vs No)**	0.996 (0.377-2.633)	0.994
**Histological (Squamous cell carcinoma vs Adenocarcinoma)**	1.095 (0.408-2.940)	0.857
**ECOG PS (3 vs 2)**	0.603 (0.052-6.996)	0.686
**ICIs type (PD1vs PD-L1)**	0.800 (0.106-6.043)	0.829
PD-L1 tumor proportion score, n (%)		
**NA**	-	-
**≥1%**	1.270 (0.383-4.210)	0.696
**<1%**	0.444 (0.118-1.667)	0.230
**With Distant metastasis (Yes vs No)**	1.421 (0.536-3.765)	0.480
**Combine other irAEs (Yes vs No)**	0.596 (0.136-2.621)	0.494
**Combine cardiovascular disease (Yes vs No)**	0.648 (0.191-2.199)	0.487
Pre-existing respiratory disease		
**COPD (Yes vs No)**	0.479 (0.131-1.744)	0.264
**Emphysema (Yes vs No)**	0.500 (0.161-1.550)	0.230
**Line (First-line vs ≥ Second-line)**	2.893 (0.707-11.843)	0.140
**Suspension of anti-tumor therapy (Yes vs No)**	3.598 (1.307-9.905)	**0.013**
Treatment of CIP (Yes vs No)		
**Immunosuppressant**	0.000 (0.000-Inf)	0.991
**Anti-fibrosis**	1.412 (0.525-3.793)	0.494
**Hormone dosage level (High vs Low)**	2.743 (0.807-9.327)	0.106
**IL-2, pg/mL**	0.992 (0.966-1.018)	0.531
**IL-4, pg/mL**	1.045 (0.915-1.194)	0.513
**IL-6, pg/mL**	1.000 (1.000-1.000)	0.399
**IL-10, pg/mL**	1.010 (0.983-1.037)	0.475
**TNF-α, pg/mL**	1.021 (0.966-1.079)	0.461
**IFN-γ, pg/mL**	0.983 (0.949-1.018)	0.343
**NEUT, K/μL**	1.018 (0.981-1.056)	0.336
**ALC, K/μL**	1.162 (0.816-1.655)	0.405
**PLT, K/μL**	1.001 (0.998-1.004)	0.587
**NLR**	1.008 (0.982-1.035)	0.535
**PLR**	0.999 (0.997-1.001)	0.487
**hsCRP**	0.998 (0.990-1.005)	0.502
**LDH, U/L**	0.998 (0.996-1.000)	0.124
**ALB, g/L**	1.028 (0.913-1.156)	0.651
**D-dimer**	1.000 (1.000-1.000)	0.838
**KL-6**	1.001 (1.001-1.002)	**<0.001**

Immunosuppressant included Infliximab; Anti-fibrosis included pirfenidone; Hormone (high): glucocorticoid dosage 30-100mg/day; Hormone (low): 7.5-30mg/day. CIP, checkpoint inhibitor related pneumonitis; OR, odds ratio; BMI, Body Mass Index; ECOG, Eastern Cooperative Oncology Group performance status; ICI, immune checkpoint inhibitor; PD-1 programmed cell death protein-1; irAEs, Immune-related adverse events; IL-2, interleukin-2; TNF-α, tumor necrosis factor; IFN –γ, interferon-gamma receptor; NEUT, neutrophil; ALC, absolute lymphocyte count; PLT, platelet; NLR, neutrophil-to-lymphocyte; PLR, platelet lymphocyte ratio; hsCRP, high sensitivity C reactive protein; LDH, lactate dehydrogenase; ALB, albumin; KL-6, human sialylated carbohydrate antigen 6.

**Table 6:** The multivariate logistic regression analysis of risk factors for poor prognosis in patients with severe CIP.

Variables	OR (95% CI)	*p*-value
**Suspension of anti-tumor therapy (Yes vs No)**	4.247 (1.067-16.915)	**0.040**
**IL-6, pg/mL**	1.000 (0.999-1.000)	0.964
**IL-10, pg/mL**	1.017 (0.970-1.066)	0.499
**LDH, U/L**	0.997 (0.995-1.000)	0.046
**KL-6**	1.002 (1.001-1.002)	**<0 .001**
**Smoking history (Yes vs No)**	1.360 (0.334-5.536)	0.667

CIP, checkpoint inhibitor related pneumonitis; OR, odds ratio; IL, interleukin; LDH, lactate dehydrogenase; KL-6 human sialylated carbohydrate antigen 6.

The bold values are meaningful p-values indicating that this factor is meaningful in the multivariate regression.

## Discussion

Accompanied by promising survival benefits, ICIs are associated with a broad spectrum of toxic effects known as irAEs, including rash, colitis, hepatitis, endocrinopathies, and pneumonitis. Another Meta-analysis revealed that both the overall incidence and the rate of severe CIP occurrence in lung cancer patients surpass those in patients with other malignancies (21, 22).

Most irAEs are mild and tolerable, some can be fatal. Severe pulmonary irAEs, notably CIP, are rare but carry a high mortality rate, often overlapping clinically and radiologically with respiratory symptoms of the primary tumor (23). Although many articles considered that the development of irAEs of any grade was significantly associated with better clinical outcomes in patients with advanced NSCLC treated with ICIs monotherapy (20, 24). However, the results of this study showed that patients with severe grade CIP had a worst prognosis than those with milder CIP, as in the previous article (9). ECOG PS scores in all patients who died due to CIP were two or worse. Moreover, many patients with severe CIP have pulmonary infections because of their poor pulmonary status may also be a risk factor for their poor prognosis.

The mortality rate of severe CIP patients in this study is relatively high. One reason is that our team started the CIP research earlier and did more research on it, with a special focus on treating immune-related adverse events, especially CIP, so we received more severe CIP patients at first diagnosis and referral. Another reason is that some patients also suffered from some basic diseases such as ILD and COPD. As a real-world study, the mortality rate is relatively high, similar to the mortality rates of 22.7% and 28% reported in two real-world studies.

Previous studies rarely reported the causes of death in patients with severe CIP. This study conducted an in-depth analysis of the patient population with severe CIP and found that The primary cause of death among all patients with severe CIP is CIP complicated with infection. However, the leading causes of mortality differ between the poorer prognosis group and the better prognosis group within the severe CIP cohort, with CIP complicated with infection being the primary cause in the PP group and tumor progression being the primary cause in the GP group. Based on this finding, we recommend further expanding the study sample to comprehensively investigate this phenomenon. This may be due to the fact that poorer prognosis group, coupled with severe infections, progresses rapidly. Although CIP was better controlled in the good prognosis group, the longer suspension of anti-tumor treatment resulted in difficult-to-control tumor progression, ultimately leading to more deaths from tumor progression. The clinicians' tendency to prioritize the treatment of CIP while overlooking antitumor therapy could be the reason behind this. Therefore, it was necessary to give patients timely anti-tumor therapy, such as continuing chemotherapy regimen or increasing anti-vascular therapy. It is also crucial to note the importance of promptly controlling infections associated with CIP.

In addition, this study showed that elevated KL-6 concentrations predicted a poor prognosis in patients with CIP. KL-6 is a high molecular weight glycoprotein encoded by Mucin gene, which is mainly distributed on the cell surface of type II alveolar epithelial cells (AECs), and the elevated KL-6 concentrations typically disrupt alveolar capillaries and type II AECs regeneration (25, 26). Articles have been reported the elevated KL-6 level indicated more severe, more progressive, and predicted the higher mortality and poor outcomes of ILD (Interstitial lung disease) (20, 24). CIP is closely associated with interstitial pneumonia (27). ICIs-associated lung injury manifests in a variety of forms, including interstitial pneumonia.

Due to insufficient preclinical research, understanding the mechanisms of CIP remains an area requiring further exploration. CIP in non-small cell lung cancer results from various contributing factors. Studies indicate that PD-1/PD-L1 inhibitors can boost T cell anti-tumor activity. Activated T cells infiltrate lung tissues in CIP patients, signaling increased anti-tumor effects. However, excessive immune responses can harm normal tissues (28–31). Past research indicates a possible link between CIP development and heightened levels of pre-existing and newly appearing autoantibodies in human immunity. Pre-existing antibodies like rheumatoid factor (RF), antinuclear antibodies, anti-thyroglobulin, and anti-thyroid peroxidase antibodies are independently associated with irAE occurrence in various organs (32, 33). Salahaldin A. Tahir and colleagues additionally discovered a significant 1.34-fold increase in autoantibodies against CD74 in patients with immune-related pneumonia after receiving immune checkpoint inhibitor therapy, suggesting a role for CD74 autoantibodies in pneumonia (34). Some research indicates that the pathophysiology of irAEs may involve cytokine mediation. In a study with melanoma patients, certain cytokines like G-CSF, GM-CSF, Fractalkine, FGF-2, IFNα2, IL-12p70, IL-1α, IL1, IL-1RA, IL-2, and IL-13 showed significant upregulation at baseline and early treatment stages, correlating with high-grade irAE occurrence (35)In a study of NSCLC patients receiving ICIs treatment, interleukin-6 (IL-6), IL-17A, IL-35, C-reactive protein (CRP), procalcitonin (PCT), surfactant protein-D (SP-D), and Krebs von den Lungen-6 (KL-6) were more frequently observed in patients with CIP compared to those without CIP (36). Several other potential mechanisms remain to be explored, with some studies suggesting that the modulation of the gut microbiome may be associated with the efficacy and toxicity of immunotherapy (37, 38) Hakozaki and colleagues noted notable distinctions in the gut microbiomes of late-stage NSCLC patients who developed low-grade versus high-grade irAEs (39). Further exploration is warranted to elucidate the involvement of non-coding RNAs, such as microRNA-146a, in regulating irAEs (40). The associated mechanisms of severe CIP and its relationship with outcomes necessitate additional investigation.

There were some limitations to our study. First, this was a single-center retrospective study with a limited sample size, so information bias cannot be ruled out. Another limitation was that we did not exclude differences in the tumor histological type and ECOG score when comparing OS in the severe CIP group and mild CIP groups, which may affect the validity of the final results. Nevertheless, we reported a strong relationship between CIP grade and prognosis, associated risk factors, and leading causes of death, which have some clinical significance. In follow-up studies, we will conduct a prospective study with a large sample size to explore prognostic risk factors in patients with CIP.

## Conclusions

In conclusion, patients with severe CIP have a poor prognosis, especially those with elevated KL-6, and the main cause of death is immune checkpoint inhibitor-associated pneumonitis complicated with infection. In addition, anti-tumor therapy for severe CIP patients should be resumed in time and should not be delayed for too long.

## Data availability statement

The original contributions presented in the study are included in the article/supplementary material. Further inquiries can be directed to the corresponding authors.

## Ethics statement

The studies involving humans were approved by the local Ethics Committee of the First Affiliated Hospital of Guangzhou Medical University. The studies were conducted in accordance with the local legislation and institutional requirements. The participants provided their written informed consent to participate in this study.

## Author contributions

NS: Data curation, Formal analysis, Investigation, Writing – original draft. RL: Data curation, Formal analysis, Investigation, Writing – original draft. HD: Data curation, Formal analysis, Investigation, Writing – original draft. QL: Software, Writing – original draft. JD: Supervision, Writing – original draft. YZ: Validation, Writing – original draft. WM: Visualization, Writing – original draft. WG: Investigation, Writing – original draft. MH: Data curation, Writing – original draft. ML: Software, Writing – original draft. XX: Visualization, Writing – original draft. XL: Conceptualization, Methodology, Project administration, Writing – review & editing. CZ: Conceptualization, Funding acquisition, Methodology, Project administration, Writing – review & editing.
